# Mindfulness-Based Stress Reduction in Breast Cancer Survivors with Chronic Neuropathic Pain: A Randomized Controlled Trial

**DOI:** 10.1155/2022/4020550

**Published:** 2022-07-07

**Authors:** Yaadwinder Shergill, Danielle B. Rice, Eve-Ling Khoo, Virginia Jarvis, Tinghua Zhang, Monica Taljaard, Keith G. Wilson, Heather Romanow, Brittany Glynn, Rebecca Small, Joshua A. Rash, Andra Smith, Lynette Monteiro, Catherine Smyth, Patricia A. Poulin

**Affiliations:** ^1^The Ottawa Hospital Research Institute, Ottawa, Ontario, Canada; ^2^One Elephant Integrative Health Team, Oakville, Ontario, Canada; ^3^Department of Health Research Methods Evidence and Impact, McMaster University, Hamilton, Ontario, Canada; ^4^Department of Psychology, The Ottawa Hospital, Ottawa, Ontario, Canada; ^5^Department of Anesthesiology and Pain Medicine, Faculty of Medicine, University of Ottawa, Ottawa, Ontario, Canada; ^6^Department of Psychology, Memorial University of Newfoundland, St. John's, NL, Canada; ^7^Department of Psychology, University of Ottawa, Ottawa, Ontario, Canada; ^8^Ottawa Mindfulness Clinic, Ottawa, Ontario, Canada; ^9^Department of Anesthesiology and Pain Medicine, The Ottawa Hospital, Ottawa, Ontario, Canada

## Abstract

**Objectives:**

The purpose of this study was to compare the effects of group-delivered mindfulness-based stress reduction as compared to a waitlist control group among breast cancer survivors living with CNP.

**Methods:**

A randomized controlled trial design was applied, and outcomes collected included pain, emotional function, quality of life, and global impression of change.

**Results:**

A total of 98 women were randomized and included in analyses. The sample included 49 women in the mindfulness-based stress reduction group, and 49 women in the waitlist control group. The intervention group participants (mean age 51.3 years, standard deviation = 11.4) and waitlist participants (mean age 55.1 years, standard deviation = 9.6) reported an average pain duration of approximately three years. No significant differences were found on the primary outcome of the proportions of women with reduced pain interference scores from the time of randomization to 3 months after the intervention was received. No significant changes were found among secondary outcomes.

**Conclusion:**

Our randomized clinical trial did not find significant benefits of group-based mindfulness-based stress reduction for the management of CNP. The current study findings should be replicated and are important to consider given ongoing concerns that nonsignificant results of mindfulness-based stress reduction are often unpublished.

## 1. Introduction

Breast cancer is the most commonly occurring cancer among women worldwide [1]. Although the incidence and prevalence of breast cancer have increased during the past decade, mortality rates have declined due to early diagnosis and improved treatments [1, 2]. This has resulted in increasing numbers of women living with long-term sequelae of the disease or its treatments. Chronic neuropathic pain (CNP) resulting from tumor infiltration, surgery, chemotherapy, or radiation therapy affects between thirty to fifty percent of breast cancer survivors [3]; it is often severe and hard to treat [4], resulting in disability and reduced health-related quality of life among breast cancer survivors [1].

The Canadian Pain Society consensus statement for the pharmacologic management of CNP recommends gabapentinoids, tricyclic antidepressants, and serotonin-norepinephrine reuptake inhibitors as first-line agents [5]. Many individuals with CNP continue to report disabling pain [6] and associated psychological distress despite optimal medical management [7]. Consequently, interventions that address the psychological factors that may influence pain experience are often recommended [8]. However, the effectiveness of psychosocial approaches in the management of chronic neuropathic pain is not known [1].

Mindfulness-based stress reduction (MBSR) is an evidence-based and group-based intervention that focusses on improving awareness and acceptance of thoughts and feelings, including physical discomfort and difficult emotions [9]. The core components of MBSR consist of meditation practices that serve to (1) increase awareness of sensations, emotions, and thoughts; (2) provide self-regulation strategies; and (3) promote healthy and adaptive responses to stress [10]. Multiple reviews highlight the benefits of MBSR among patients with cancer [11–15] and chronic pain [16, 17]. For example; a meta-analysis of 10 randomized controlled, and observational studies including 583 patients reported that interventions involving mindfulness in cancer care are associated with positive mental and physical health outcomes [11]; a systematic review of 25 studies involving 1,285 patients with chronic noncancer pain found that patients who completed mindfulness-based interventions reported improvements in pain intensity, depression, anxiety, physical wellbeing, and quality of life, with medium effect sizes [18]. However, none of the primary studies included in these reviews looked at the effects of mindfulness for CNP in breast cancer survivors. To our knowledge, there is only one primary study that has focused on CNP; a study with people living with painful peripheral diabetic neuropathy found that MBSR resulted in reduced pain-related disability and severity among several other benefits in comparison to a waitlist control group [19].

The purpose of our study was to determine the effects of MBSR among breast cancer survivors living with CNP after ensuring stability of pharmacological management. Our primary hypothesis as reported in a registered protocol on ClinicalTrials.gov was that, completion of group MBSR would result in at least 30% more women achieving *a* ≥ 1.0 point improvement in pain-related interference on the Brief Pain Inventory at 3-month follow-up [20] in the intervention group, relative to the waitlist control group. Our secondary hypotheses were that participation in MBSR would result in reduced psychological distress and pain-related cognition, and improved quality of life at 3-month follow-up relative to control. Three exploratory studies focusing on the effects of MBSR training on cognitive function [21], brain structure [22] and function [23], and biomarkers of stress and inflammation [24] are reported in detail in separate manuscripts.

## 2. Methods

The study protocol was prepared with consideration of the CONSORT 2010 Statement: Updated guidelines for reporting parallel group randomized trials [25], approved by the institutional research ethics board and registered on clinicaltrials.gov (NCT02758197) prior to participant recruitment. The study began prior to the publications of The CONSORT Statement for social and psychological interventions [26] (CONSORT-SPI 2018), but we have included the checklist as a supplementary item.

### 2.1. Experimental Design

This is a randomized waitlist controlled study evaluating the effects of MBSR to waiting following consultation with a pain specialist for medical optimization and confirmation of pharmacological treatment stability prior to randomization to the treatment arms. Enrollment of study participants began January 2014 with the last follow-up conducted in November 2017.

### 2.2. Population

We recruited 144 female breast cancer survivors with CNP who were at least 1-year post-treatment of cancer, had neuropathic pain for more than 6 months in duration, and a baseline pain severity score of 4 or greater (moderate to severe) on the Brief Pain Inventory-Severity Scale. Diagnosis was confirmed by a pain specialist prior to entry into the study. To be eligible, participants had to be able to complete questionnaires in English or French and participate in mindfulness training sessions conducted in English. Participants also had to be able to commit to attending a minimum of 7 (out of a possible 9) weekly MBSR sessions. Participants were excluded if they had prior experience with MBSR, had an expected survival of less than 12 months, had cognitive impairment, or severe psychiatric disorder impacting their ability to participate (e.g., schizophrenia).

### 2.3. Procedures

Prospective participants were recruited through poster advertisements and health care teams at a tertiary health science centre and at multiple regional institutions providing services to people affected by cancer throughout the region. Individuals interested in the study underwent screening with a research staff prior to being seen for consultation by a pain specialist for a consultation. The objectives of this consultation were to (1) confirm the diagnosis of neuropathic pain and (2) optimize pharmacological management of pain. Participants who were prescribed medications or provided with recommendations to discuss with their family physicians were required to be stable on their medication for a minimum of 2 weeks prior to randomization and this was verified during a brief phone interview with the participant by the study research coordinator. This was done to determine the effects of MBSR under circumstances that paralleled best practices in usual care. We collected the outcome data for both groups at 4 time points: T1: before medical optimization; T2: after randomization; T3: 2 weeks post-MBSR (and equivalent time point for control group); and T4: 3 months post-MBSR (and equivalent time point for control group). The waitlist participants were offered MBSR after the 3-month follow-up.

### 2.4. Randomization and Blinding

Participants who were on a stable medication regiment for a minimum of 2 weeks were randomly assigned to either the MBSR or waiting arm. Simple stratified allocation procedures were applied with participants being allocated randomly in strata to one of the two study arms [27]. Participants were stratified on two conditions: (1) pain due to postmastectomy or pain from chemotherapy-induced peripheral neuropathy and (2) pain severity categorized as 4–6 (moderate pain) and 7–10 (severe pain) as these are important prognostic variables [28]. Allocations were performed by a statistician who was not associated with the study using computer-generated, stratified, and permuted block design with randomly varying block lengths of 2, 4, or 6. Only the research coordinators (HR, YS, and EKL) were aware of treatment assignments. Allocations were concealed from investigators, treating physicians, statisticians, and research assistants. Participants were assessed by research assistants who had no knowledge of group allocations and were asked explicitly to not discuss their group allocation with the research assistants.

### 2.5. Interventions

The intervention followed the MBSR format, with minor modifications by some of the group facilitators (e.g., different mindful movement practices and more emphasis on clarifying personal values). It consisted of eight, 2.5-hour weekly sessions along with a full day (approximately 6 hours) retreat held halfway through the course on a weekend. Each group included approximately 8–12 participants. Courses were coordinated by the research team, specifically for the study, and held at the Ottawa Regional Cancer Foundation. In some cases where participants were unable to attend a group arranged by the research team, they were registered into one of the MBSR programs for people living with various difficulties offered in the community by other healthcare providers. MBSR courses were offered throughout the region, allowing for participants to choose a convenient location and time. Each session included time dedicated to mindfulness meditation practice, discussion about participant experience of meditation, and applications of mindfulness in daily life. In addition, the groups discussed specific themes such as stress, pain, personal attitude and values, and mindful communication. Sessions were conducted by healthcare professionals (psychologists and social workers) with formal certification in MBSR and reported a minimum of 5 years of experience providing MBSR in a group format. Two exceptions were made to accommodate participants' scheduling needs, where one facilitator who was a postdoctoral student certified to teach mindfulness-based interventions, and another was a graduate student with more than 5 years of experience with mindfulness-based interventions and two years of teaching experience who cofacilitated sessions. Both were supervised by a healthcare provider with MBSR experience. All waitlist participants were offered participation in the MBSR program following conclusion of the waiting period. Participants were compensated $25 for each study visit and were reimbursed for the cost of parking.

### 2.6. Measures

The *primary outcome* was the average score of the interference subscale of the Brief Pain Inventory Short Form at 3-months [29]. “There are 7 numerical pain interference scales of the BPI, with each scale scoring from 0 (does not interfere) to 10 (completely interferes) in relation to general activity, mood, walking ability, work/school, mood, relationships with other people, sleep, and enjoyment of life [29].” The BPI scores provide more useful information about the patient outcome and pain-related disability than pain severity scores alone [30] and is one of the core outcomes recommended by the Initiative of Methods, Measurement, and Pain Assessment in Clinical Trials (IMMPACT) group [31]. We defined a responder as a participant who showed a decrease of ≥1.0 on this measure which represents a minimally clinically important change [32]. We tested the effect of the intervention by comparing the proportions of responders between the two arms measured at 3-month follow-up (T4) compared to prerandomization (T2).

The secondary outcomes included pain, emotional function, quality of life, and global impression of change as informed by the IMMPACT group [31]. Adverse events could include, but were not limited to, medication side effects, and increased anxiety.

*The Neuropathic Pain Symptom Inventory* (NPSI) is a 12-item patient-reported questionnaire. The NPSI is rated on a numerical scale (0–10) resulting in a total intensity score. In addition, five subscores are calculated with the following categories: “spontaneous burning pain, spontaneous pressing pain, paroxysmal pain, evoked pain, and paresthesia/dysesthesia [33].” The NPSI total score was used in this study and has been shown to be reliable, valid, and sensitive to the effects of treatment [33].

*The Patient Health Questionnaire*-*9* (PHQ-9): the PHQ-9 was used to evaluate depressive symptoms. This 9-item scale assesses the severity of depressive symptoms over the past two weeks and is based on DSM-IV diagnostic criteria for major depression [34]. This questionnaire is scored from 0 to 27, with clinical cut-points indicative of mild, moderate, moderately severe, and severe depression [34]. A 5-point decrease on the PHQ-9 is considered to be the minimum clinically significant change [35].

*The Pain Catastrophizing Scale* (PCS): the PCS is a validated, patient-reported 13-item instrument which quantifies an individual's negative thoughts when in pain [36]. There are three subscales of the PCS: rumination, magnification, and helplessness. Each item is rated using a Likert scale (0 = not at all, 4 = all the time). The psychometric values of the PCS are well-documented and suggest good reliability (total score = *α* = 0.86), test-retest stability, and concurrent, criterion-related, and discriminant validity [15, 36].

*Five Facet Mindfulness Questionnaire* (FFMQ): the FFMQ is a 39 question instrument reporting on five aspects of mindfulness: “nonreactivity to inner experience, observing, describing, acting with awareness, and nonjudging of experience [37].” Participants are asked to use a five-point Likert-type scale to rate how true of them they believe each statement to be. The FFMQ has been found to have adequate to good reliability, with alpha coefficients ranging from 0.75 to 0.91 for all subscales. Mindfulness total score explains a significant proportion of the variance in pain catastrophizing, pain-related fear, pain hypervigilance, and disability in breast cancer survivors living with chronic neuropathic pain [38].

*Short-Form-12 Health Survey* (SF-12v.2): the SF-12v.2 is a brief, twelve-item self-report measure of health-related quality of life. This measure is based on the widely used and empirically validated Short-Form-36 [39, 40]. One of the primary advantages of the SF-12 is that it takes only a few minutes to complete. The SF-12 assesses eight health domains including “bodily pain, social functioning, physical functioning, vitality role limitations due to physical health and emotional health, and general and mental health.” A physical composite scale (PCS) and mental composite scale (MCS) are calculated from 12 items. The SF-12 is scored from 0 to 100, with higher scores indicating greater health-related quality of life.

*Profile of Mood States* (POMS): the POMS is composed of 37 questions, rated on a five-point scale ranging from “not at all” to “extremely,” assessing transient, distinct mood states. The POMS scale can measure six different dimensions of mood swings including “anxiety-tension, anger-hostility, vigor-activity, fatigue-inertia, dejection-depression, and confusion-bewilderment, over a period of time [41].”

*Patient Global Impression of Change* (PGIC): the PGIC is a one item self-report questionnaire that measures a patient's belief about the efficacy of the treatment using a seven-point scale ranging from “very much improved” to “very much worse.” This scale is widely used in chronic pain trials; however, the validity has not been addressed [42].

*Perceived Stress Scale (PSS)*: The PSS consists of 10 questions, ranging on a five-point scale ranging from “never” to “very often,” measuring the perception and impact of stress over the past month with higher scores representing greater stress [43]. The PSS has been used among individuals with pain and the psychometric properties of the tool are well-documented and demonstrate good reliability, internal consistency, and discriminant validity [43–46].

*Adverse events*: adverse events were recorded by the research assistant during study visit follow ups.

### 2.7. Demographic and Medical Characteristics

Variables such as level of education, employment status, marital status, and medical history were recorded. A 2-year medication history was also taken at baseline as well as at 3-month follow-up and changes were determined based on pharmacy records where medication was coded as being (1) unchanged, (2) increased, (3) decreased, or (4) undetermined during the study period.

### 2.8. Treatment Fidelity

A registered psychotherapist with more than 5 years of experience with mindfulness-based interventions assessed treatment fidelity [47] by listening to a random selection of 12% of the session recordings. The Bangor, Exeter and Oxford Mindfulness-Based Interventions: Teaching Assessment Criteria Scale (MBI:TAC) [48] and adherence component of the Mindfulness-Based Relapse Prevention Adherence and Competence Scale [49] were used by the reviewer to rate the extent to which the facilitator adhered to the following domains: “(1) organization of session curriculum; (2) relational skills; (3) embodiment of mindfulness; (4) guiding mindfulness practices; (5) conveying course themes through interactive inquiry and didactic teaching; and (6) holding of group learning environment.” The reviewer noted competency level for the facilitator using a Likert scale (1 = incompetent to 6 = advanced).

### 2.9. Data Analysis

Our required sample size was calculated as 36 and 57 participants in the control and intervention arms, respectively. This calculation was performed a-priori and used methods appropriate for partially nested trials comparing groups and individuals. The calculations were based on 80% power at a two-sided 5% significance level and used the following assumptions: (a) control arm proportion of respondents of 0.20; (b) experimental arm proportion of respondents of 0.50; (c) intracluster correlation coefficient (ICC) = 0.05; and (d) an average of 13 participants per MBSR group. Our target difference was 30%, which was considered the smallest difference that would encourage us to pursue a later comparative study. To account for attrition (15% or no more than 3 participants per group), we planned to enroll 44 participants (control arm) and 64 participants (intervention arm).

Descriptive statistics were calculated to compare baseline and preintervention demographic and clinical characteristics between the intervention and waitlist control arms. Mean and standard deviation were calculated on continuous variables. Frequencies and proportions were calculated for categorical variables.

The data were analyzed for the primary outcome using mixed effects logistic regression with group indicated as a random effect to account for the partially nested design. The main independent variable was treatment allocation. Covariates entered into statistical models were defined a-priori and included the stratification factors BPI pain severity at baseline and pain etiology (postmastectomy vs. chemotherapy-induced). The treatment effect was expressed as an adjusted odds ratio (OR) with 95% confidence interval (CI).

To examine the potential impact of the missing data on the results, we conducted a sensitivity analysis for the primary outcome by first imputing all participants with missing outcomes as nonresponders and then as responders [50]. We also analyzed the primary outcome (BPI-Interference score) as a continuous rather than a dichotomous variable, allowing us to maintain the integrity of the scale. For the continuous primary and secondary outcomes, mixed effects linear regression was used to account for the partially nested design allowing for a heterogeneous variance structure [51] that accounted for the correlation in repeated measures using a compound symmetric covariance structure. The Kenward–Roger method was used to calculate degrees of freedom [52]. Study visit, arms, and the interaction between visit and arm were included in the models to calculate the difference between the intervention and control arms over time. Least square means and the mean changes from the time of randomization to 2 weeks and 3 months after intervention in each group with 95% CI were obtained along with between-arm least square mean difference in change from baseline to 3 months. Analyses were conducted using SAS v.9.4 (SAS Institute Inc., Cary, NC, USA) [53].

## 3. Results

**Figure 1:**
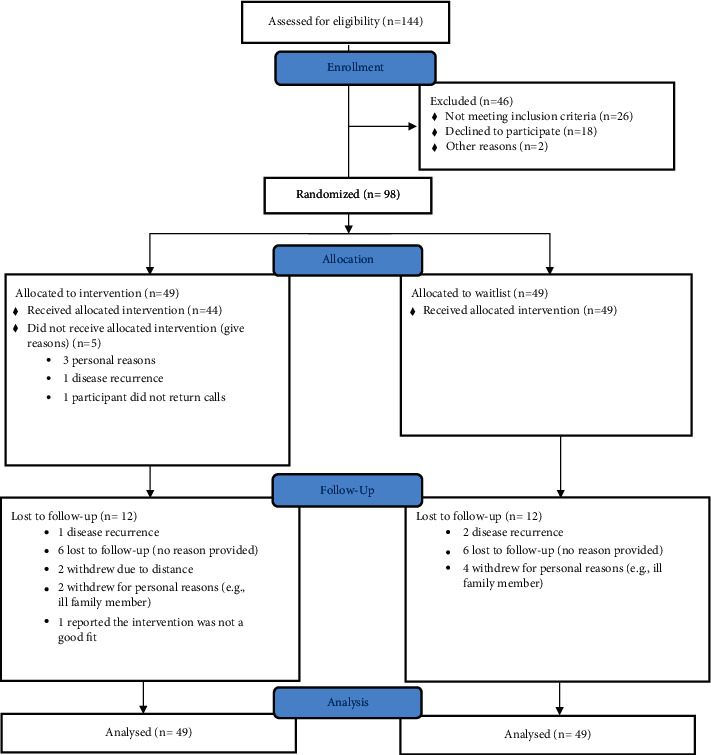
Consort study flow.

Study flow is depicted in Figure 1. In total, 98 out of 144 women assessed for eligibility met inclusion criteria and were randomized to receive MBSR (*n* = 49) or waiting (*n* = 49). Thirty-seven individuals (76%) in each group completed the study, with 12 (24%) participants lost to follow-up in each group. Thirty-one of the 49 subjects (63%) allocated to MBSR attended at least six of the eight sessions. Twenty-nine of the subjects allocated to waiting eventually received MBSR.

Participant demographics are summarized in Table 1. MBSR participants (mean age 51.3 years, standard deviation = 11.4) and waitlist participants (mean age 55.1 years, standard deviation = 9.6) reported an average pain duration of approximately three years; 40 (40.8%) reported primarily chemotherapy-induced peripheral neuropathy and 58 (59.1%) reported primarily postmastectomy pain. At least one third in each group reported full-time employment status. No significant differences were identified between the study arms at baseline.

**Figure 2:**
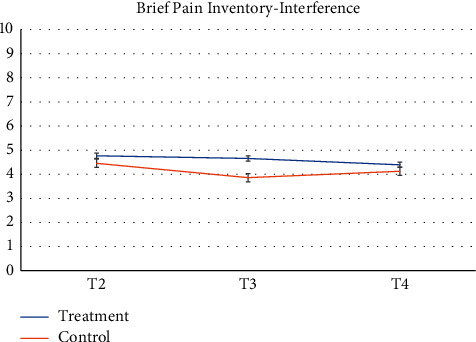
Brief Pain Inventory-Interference.

**Figure 3:**
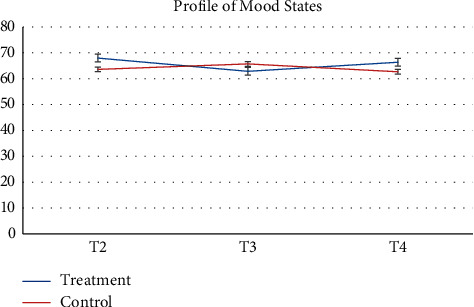
Profile of Mood States.

**Figure 4:**
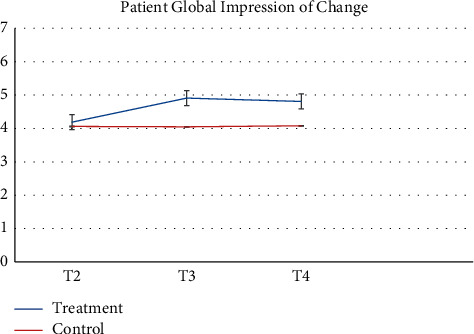
Patient Global Impression of Change.

**Figure 5:**
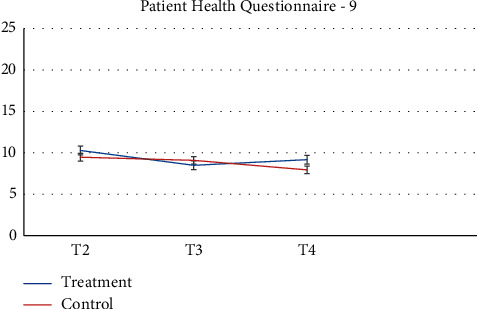
Patient Health Questionnaire-9.

**Figure 6:**
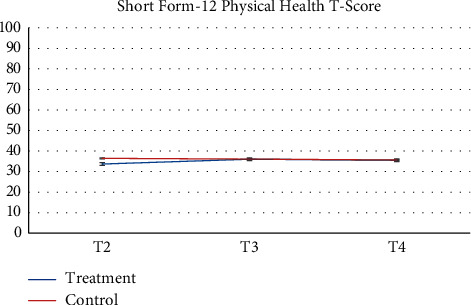
Short-Form-12 Health Survey-physical health T-score.

**Figure 7:**
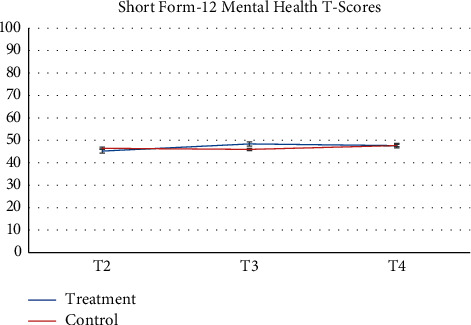
Short-form-12 Health Survey-mental health T-score.

**Figure 8:**
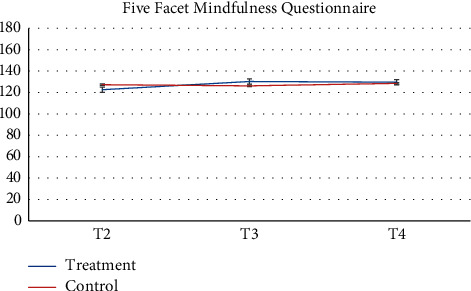
Five Facet Mindfulness Questionnaire.

**Figure 9:**
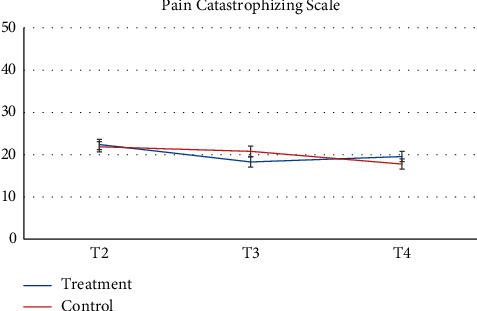
Pain Catastrophizing Scale.

**Figure 10:**
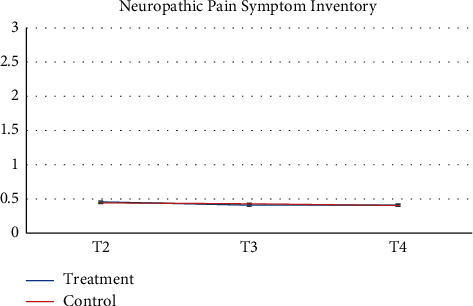
Neuropathic Pain Symptom Inventory.

Pharmacy records at baseline and 3-months follow-up were available. 64 participants and more than half of participants (*n* = 52, 81.2%) had no changes in their medication during the study. Despite the recommendation to stay on a stable medical regimen, 8 (12.5%) had their medication increased and 4 had their medications reduced (6.25%). There was a greater proportion of participants in the control group that increased their reliance on medication during the study.  Table 2 presents raw observed mean and standard deviation scores at each time point, while Figures 2–10 display them graphically.

### 3.1. Primary Outcome

The primary outcome was available for 31 participants in the treatment group and 39 in the control group. No significant differences were observed in the proportions of responders on the BPI interference scale from the time of randomization (T2) to 3 months after the MBSR course ended (T4) between participants in the treatment group (*N* = 11, 35.5%) as compared to the control group (*N* = 8, 20.5%) (adjusted OR 1.96, 95% CI 0.60 to 6.41, *p* = 0.2633) (Table 3).

Sensitivity analyses that accounted for the missing data produced similar results.

### 3.2. Secondary Outcomes

Least square mean scores prior to the intervention delivery (T2), least square mean changes within each group, as well as between-group differences in change scores at the primary endpoint of 3 months obtained from the linear mixed effects regression analyses are provided in Table 4. No significant between-group differences in change of the scores (T4-T2) were observed on any subscale. BPI scores were largely unchanged between each time points for both groups. When analyzed as a continuous variable, the least square mean change in the BPI score from the time of randomization (T2) to 3 months (T4) in the treatment group was −0.33 (95% CI −0.96 to 0.30) compared to −0.37 (95% CI −0.91 to 0.17) in the control group (between-arm difference 0.04, 95% CI −0.97 to 0.86, *p* = 0.93). Participants reported the most notable change across time on the POMS, whereby the treatment group demonstrated nonsignificant improvements at T3 while the control group had nonsignificant deterioration at T3. POMS scores at T4 were similar to baseline values among both groups with no significant difference between groups. Participants demonstrated nonsignificant improvements on the SF-12 mental health and physical health *t*-scores at T3 for the MBSR group with minimal changes occurring in the control group at this time point. At T4, the treatment group improvements had decreased while the control group demonstrated slightly improved mental health scores but few changes for the physical health score. Reports of depressive symptoms (PHQ-9), pain catastrophizing (PCS), and neuropathic pain (NPSI) were comparable between groups and across time points with minor reductions in symptoms present for the treatment group at T3 but a return to baseline by T4. Similarly, reports of mindfulness (FFMQ) were comparable between groups and across timepoints with minor improvements in symptoms present for the treatment group at T3 but a return to baseline by T4. The treatment group reported a slightly increased global impression of change at T3 and T4 as compared to the control group. At the 3-month follow-up (T4), participant's global impression of change scores were rated as “very much improved” or “much improved” for 9 of the MBSR groups and 9 for the control group members, respectively. No participants reported adverse events during the study trial.

### 3.3. Treatment Fidelity

The mean MBI:TAC score (average across 8 recordings) was 5.49 (SD = 0.27, range 5.0–5.75). The means and standard deviations for the individual domains were 5.75 ± 0.43 (coverage/organization), 5.75 ± 0.43 (relational skills), 5.6 ± 0.48 (embodiment), 5.6 ± 0.48 (guiding practices), 5.25 ± 0.82 (inquiry and teaching), and 5 ± 0.7 (group facilitation). This suggests that the competence scores of the reviewed sessions were rated on average as ‘proficient' level.

## 4. Discussion

This study randomized 98 breast cancer survivors with CNP to an 8-week group MBSR intervention or a waitlist control group following a pain medicine consultation and ensuring a stable pharmacological management to determine the impact of MBSR pain-related interference, quality of life, and mental health outcomes. We found that a larger proportion of participants from the MBSR group improved over the course of the study on our primary outcome of pain-related disability, but the confidence interval around this difference was wide and included the null value. Although slight and more rapid improvement in pain intensity were noted in the MBSR group in the short term (2-week follow-up), no significant between-group differences were found for any of the self-report measures assessed at 3-months follow-up after controlling for preintervention pain intensity.

These results are unexpected. Firstly, several other studies have demonstrated that MBSR is effective in reducing symptoms associated with breast cancer or its treatment [11, 14, 54–56] including cancer-related pain [18, 39]. Secondly, our exploratory neuroimaging studies investigating the influence of mindfulness on brain resting state and white matter integrity as well as emotional reactivity among 21 participants found a potential impact of MBSR on the neural correlates of CNP due to breast cancer treatment. These included improved white matter integrity in several brain regions [23], reduced activation in several brain areas involved in pain processing and emotional processing in the presence of pain-related stimuli (emotional Stroop task) [22], as well as altered default mode network activity/connectivity post-MBSR in comparison to controls [21]. The effect of natural recovery from cancer treatment-induced chronic neuropathic pain may have masked any effect of MBSR in our population. Indeed, there is a variable course for natural recovery along with multiple treatment options for neuropathic pain due to its heterogeneous makeup [57].

Given that there is considerable evidence of the positive effects of MBSR in chronic noncancer pain [26, 58, 59], the findings of this study suggest that the response to MBSR may differ based on pain phenotypes and timing of the intervention in the disease trajectory. Focusing on neuropathic pain, a previous study of patients with painful peripheral diabetic neuropathy who completed MBSR found clinically and statistically significant improvements in pain-related disability, pain severity, and mood symptoms [19]. Furthermore, MBSR was more effective for people who had lived with diabetes the longest [25]. It may be that individuals who have lived with a chronic disease the longest have exhausted various treatment options and are more likely to benefit from an approach that privileges acceptance and peaceful coexistence with pain and other symptoms. In order to guide clinical research, future MBSR on chronic pain needs to take into account/address pain, medical, and patient characteristics that moderate/mediate treatment effect.

It is also important to consider that the conferred benefits of MBSR in our study, if there are any, are not identified through the key outcomes typically measured in chronic pain treatment trials; several of our study participants spontaneously provided very positive feedback about their participation in the program, with many commenting that they wished they had had access to it during their active cancer treatment. While consensus on core outcomes for pain clinical trials focus on reductions in pain, disability, and psychological distress, exploring how participants' relationship to pain, distress, and disability changes over time throughout participation in mindfulness-based interventions maybe worthwhile as this is more closely aligned with the intention of the practice. This will require careful consideration of the value placed on various outcomes. As Kabat-Zinn puts it, “What if this is as good as it gets.” Future studies could also focus on psychological features that may moderate treatment effects (e.g., personality attributes such as openness to experience, readiness for change, or chronic pain acceptance). Qualitative inquiry into participants' experience may also be incorporated into future mindfulness studies to better understand the process of change, barriers and facilitators, which our team recently initiated [60].

The current study findings are important given reviews that have highlighted concern that nonsignificant results of MBSR are often unpublished [61], contributing to the file-drawer effect and resulting in an evidence base that likely overstates the true effect and efficacy of MBSR. A lack of reporting of both positive and nonsignificant trials can complicate the understanding of intervention effectiveness and can result in wasted resources [62]. MBSR is a relatively low-cost, evidence-based, nonpharmacological intervention for addressing mental and physical health [63]. However, the effectiveness of MBSR may vary across different medical conditions or patient presentations. The results from this study suggest that MBSR may not reduce pain-related disability or improve quality of life among women with CNP following breast cancer more than waiting. CNP in cancer is known to be difficult to treat and is refractory to many pharmacological treatments, and it is possible that this extends to nonpharmacological interventions as well. In this sense, CNP may be a particularly difficult problem to relieve by any means.

There are some limitations to consider when interpreting the findings of this study. First, although we asked participants to remain on a stable medical regimen, some participants had their medications adjusted where needed. Review of pharmacy records demonstrated that 10% of participants in the MBSR and 27% of participants in the control group had a change in their prescription of analgesic medications during the study period, and others may have made changes that were not available in pharmacy records. Pharmacological cointervention could influence the results and future trials may need tighter controls of pain-related pharmacotherapy, which may be difficult to implement given the nature of pain. Second, we did not collect measures of homework compliance/MBSR utilization. Therefore, the extent to which MBSR participants engaged in home practice of meditation is unknown and future trials should collect this information. Third, our recruitment target of 93 participants for the study was lower than expected, and results were subject to attrition, impacting the power of our analyses. Also, it was logistically quite challenging to create groups specific to this study. There were often scheduling conflicts and delays for participants that resulted in delays for entry into groups, and some participants entered groups that were offered in the community. While this augments the face validity and pragmatic value of our study, this may also have had an impact on treatment effectiveness. Fourth, participants were not explicitly discouraged from independently seeking mindfulness-based information whether online or in the community. It is possible that some participants in the waitlist control group did so. Lastly, participants were recruited through poster advertisements and through multiple institutions providing services to people impacted by cancer. Recruitment bias may have occurred due to varying levels of motivation and treatment expectation among participants [64]. This would reflect clinical practice, but future studies should pay careful attention to motivation and treatment expectation [65] as a critical component of the therapeutic outcomes [66]. Early in the chronic pain treatment trajectory, many patients continue to hope for a cure to their pain; the process of acceptance is dynamic and may be impacting pain perception and psychological distress levels as well as disability [1]. Conducting MBSR trials among similar participant groups in other locations may be important to understand if regional differences impact findings. Independent replication of these findings is needed to augment our confidence in our conclusion.

Despite preliminary evidence that trait mindfulness is correlated with more positive health and mental health outcomes among breast and gastrointestinal cancer survivors living with CNP pain [13], our randomized clinical trial did not find significant benefits of MBSR for the management of CNP.

## Figures and Tables

**Table 1:** Demographic characteristics at baseline.

Variable	Treatment group	Waitlist group
	(*n* = 49)^∗^	(*n* = 49)^∗^
	M (SD) or *n* (%)	M (SD) or *n* (%)
Female (%)	49 (100)	49 (100)
Age	51.3 (11.4)	55.1 (9.6)
Years since pain began	2.9 (2.0)	3.0 (1.9)
Ethnicity		
Caucasian (%)	40 (83.3)	40 (88.9)
African (%)	1 (2.0)	4 (8.9)
Asian (%)	2 (4.2)	0 (0.0)
First nations (%)	1 (2.1)	1 (2.2)
Other (%)	4 (8.3)	0 (0.0)
Employment status		
Full-time employed (%)	16 (32.7)	17 (34.6)
Part-time employed (%)	3 (6.1)	7 (14.3)
Unemployed (%)	8 (16.3)	4 (8.2)
Other (%)	22 (44.9)	18 (39.1)
*Baseline measures*		
Brief Pain Inventory-Interference	4.6 (2.4)	4.3 (2.4)
Brief Pain Inventory-Intensity	4.8 (1.7)	4.7 (1.9)
Profile of Mood States	67.4 (15.0)	62.7 (13.7)
Patient Health Questionnaire-9	11.0 (7.1)	8.8 (5.1)
Short-Form-12 Health Survey-physical health T-score	32.5 (10.2)	35.2 (10.4)
Short-Form-12 Health Survey-mental health T-score	43.1 (12.1)	46.6 (11.0)

**Table 2:** Unadjusted mean and standard deviations scores at each time point.

Measured time point	Treatment group mean (SD)	Control group mean (SD)
Brief Pain Inventory-Interference		
T1	4.60 (2.39)	4.28 (2.40)
T2	4.32 (2.37)	4.08 (2.39)
T3	3.40 (2.34)	3.92 (2.61)
T4	3.49 (2.09)	3.58 (2.61)
Profile of Mood States		
T1	67.42 (14.97)	62.72 (13.68)
T2	65.04 (15.44)	63.21 (14.8)
T3	61.42 (15.02)	63.37 (14.89)
T4	62.88 (16.67)	60.92 (16.46)
Patient Global Impression of Change		
T1	N/A	N/A
T2	4.29 (1.14)	4.24 (.94)
T3	5.11 (.71)	4.14 (1.15)
T4	5.06 (.98)	4.40 (1.22)
Patient Health Questionnaire-9		
T1	9.36 (5.99)	10.48 (6.54)
T2	9.15 (5.71)	9.54 (5.71)
T3	9.49 (5.25)	8.05 (5.12)
T4	7.61 (4.73)	7.85 (5.34)
Five Facet Mindfulness Questionnaire		
T1	122.35 (21.72)	127.91(20.67)
T2	125.43 (22.75)	130.04 (22.81)
T3	132.19 (20.20)	130.56 (22.37)
T4	132.94 (23.55)	132.98 (22.79)
Pain Catastrophizing Scale		
T1	21.69 (11.77)	19.26 (12.16)
T2	20.09 (10.57)	19.19 (11.54)
T3	15.00 (11.08)	17.60 (11.81)
T4	16.28 (12.09)	14.73 (11.11)
Neuropathic Pain Symptom Inventory		
T1	0.40 (0.17)	0.43 (0.16)
T2	0.42 (0.17)	0.42 (0.16)
T3	0.37 (0.14)	0.39 (0.18)
T4	0.37 (0.16)	0.37 (0.17)
Short-Form-12 Health Survey-physical health T-score		
T1	32.49 (10.15)	35.19 (10.38)
T2	33.99 (9.61)	36.17 (10.27)
T3	37.59 (10.25)	35.10 (10.56)
T4	36.36 (12.22)	37.21 (12.20)
Short-Form-12 Health Survey-mental health T-score		
T1	43.07 (12.13)	46.64 (10.95)
T2	45.94 (12.78)	47.06 (11.10)
T3	48.17 (10.37)	46.69 (11.16)
T4	48.98 (10.69)	48.44 (10.96)

T1: before medical optimization; T2: after randomization; T3: 2 weeks post-MBSR (and equivalent time point for waitlist control group); T4: 3 months post-MBSR (and equivalent time point for waitlist control group).

**Table 3:** Adjusted^a^ within-arm and between-arm differences for primary and secondary outcomes from logistic and mixed effects regression analyses.

	Control group (*N* = 49)	Treatment group (*N* = 49)	Treatment effect
*Primary outcome (dichotomous)*			Odds ratio (95% CI)
Brief Pain Inventory-Interference, number of responders (*N* (%))^b^	8/39 (20.5%)	11/31 (35.5%)	1.96 (0.60, 6.41) *p* = 0.2633

*Secondary outcomes (continuous)*	Mean^∗^ (95% CI)	Mean∗ (95% CI)	Between-arm difference
			Mean (95% CI), *p* value
*Brief Pain Inventory-Interference*			
T2 (randomization)	4.45 (3.76, 5.14)	4.76 (4.02, 5.51	
T3-T2 (randomization to 2 weeks)	−0.11 (−0.64, 0.42)	−0.59 (−1.19, 0.01)	
T4-T2 (randomization to 3 months)	−0.37 (−0.91, 0.17)	−0.33 (−0.96, 0.30)	0.04 (−0.79, 0.86); *p* = 0.9312

*Profile of Mood States*			
T2 (randomization)	63.61 (54.27, 72.95)	67.99 (59.86, 76.12)	
T3-T2 (randomization to 2 weeks)	2.12 (−7.82, 12.05)	−5.12 (−10.25, 0.02)	
T4-T2 (randomization to 3 months)	−0.92 (−10.86, 9.01)	−1.62 (−6.75, 3.52)	−0.69 (−11.84, 10.45)
			*p* = 0.9023

*Patient Global Impression of Change*			
T2 (randomization)	4.06 (3.54, 4.58)	4.19 (3.60, 4.79)	
T3-T2 (randomization to 2 weeks)	−0.01 (−0.48, 0.47)	0.72 (0.13, 1.31)	
T4-T2 (randomization to 3 months)	0.02 (−0.46, 0.50)	0.62 (0.03, 1.21)	0.60 (−0.14, 1.34)
			*p* = 0.1068

*Patient Health Questionnaire-9*			
T2 (randomization)	9.48 (7.21, 11.76)	10.29 (7.53, 13.04)	
T3-T2 (randomization to 2 weeks)	−0.38 (−1.74, 0.98)	−1.79 (−3.50, −0.07)	
T4-T2 (randomization to 3 months)	−1.53 (−2.88, −0.18)	−1.11 (−2.82, 0.61)	0.42 (−1.74, 2.58)
			*p* = 0.6992

*Short-Form-12 Health Survey-physical health T-score*			
T2 (randomization)	36.45 (31.56, 41.33)	33.68 (27.78, 39.59)	
T3-T2 (randomization to 2 weeks)	−0.35 (−2.93, 2.23)	2.34 (−1.13, 5.81)	
T4-T2 (randomization to 3 months)	−0.82 (−3.40, 1.75)	1.82 (−1.62, 5.26)	2.65 (−1.53, 6.82)
			*p* = 0.2067

*Short-Form-12 Health Survey-mental health T-score*			
T2 (randomization)	46.46 (41.41, 51.52)	45.22 (39.49, 50.96)	
T3-T2 (randomization to 2 weeks)	−0.50 (−3.91, 2.91)	3.18 (−0.88, 7.23)	
T4-T2 (randomization to 3 months)	1.21 (−2.19, 4.62)	2.44 (−1.52, 6.39)	1.22 (−3.94, 6.39)
			*p* = 0.6398

*Pain Catastrophizing Scale*			
T2 (randomization)	21.86 (18.19, 25.53)	22.39 (18.44, 26.33)	
T3-T2 (randomization to 2 weeks)	−1.08 (−3.76, 1.61)	−4.12 (−6.94, −1.30)	
T4-T2 (randomization to 3 months)	−4.09 (−6.85, −1.33)	−2.84 (−5.72, 0.05)	1.26 (−2.65, 5.17)
			*p* = 0.5231

*Five Facets Mindfulness Questionnaire*			
T2 (randomization)	127.31 (119.35, 135.27)	122.6 (114.65, 130.55)	
T3-T2 (randomization to 2 weeks)	−1.10 (−4.31, 2.12)	7.68 (2.25, 13.12)	
T4-T2 (randomization to 3 months)	1.24 (−2.07, 4.54)	7.02 (1.38, 12.66)	5.78 (−0.72, 12.28)
			*p* = 0.0806

*Neuropathic Pain Symptom Inventory*			
T2 (randomization)	0.444 (0.392, 0.496)	0.458 (0.402, 0.514)	
T3-T2 (randomization to 2 weeks)	−0.015 (−0.064, 0.035)	−0.049 (−0.093, −0.004)	
T4-T2 (randomization to 3 months)	−0.039 (−0.092, 0.013)	−0.046 (−0.093, −0.00005)	−0.007 (−0.076, 0.062)
			*p* = 0.8379

T2: after randomization; T3: 2 weeks post-MBSR (and equivalent time point for waitlist control group); T4: 3 months post-MBSR (and equivalent time point for waitlist control group); ^a^Analyses were adjusted for stratification factor, pre-BPI pain, and time since entering pain clinic. ^b^*N* = 10 and *N* = 18 participants in the control and intervention arms had missing values and could not be classified for the responder analysis. ∗An increase in score indicates a benefit for the SF-12, FFMQ variables, and the PGIC score, while a decrease in score indicates a benefit for all other measures (BPI, PHQ-9, POMS, PCS, and NPSI).

**Table 4:** Exploratory analyses showing least square mean changes from baseline for participants who completed MBSR after waiting pooled with participants initially allocated to MBSR.

	Least square means	Mean change from baseline (95% CI)	*p* value
*BPI-Interference*			
Baseline	4.60	NA	NA
2 weeks	4.05	−0.54 (−1.00 to −0.09)	0.0201
3 months	4.05	−0.54 (−1.01 to −0.08)	0.0231

*POMS*			
Baseline	62.63	NA	NA
2 weeks	59.10	−6.06 (−8.76 to −3.37)	<0.0001
3 months	56.56	−3.53 (−6.27 to −0.80)	0.013

*PGIC*			
Baseline	4.14	NA	NA
2 weeks	4.65	0.76 (0.34 to 1.19)	0.001
3 months	4.90	0.52 (0.08 to 0.94)	0.0208

*PHQ-9*			
Baseline	8.90	NA	NA
2 weeks	7.60	−0.98 (−1.92 to −0.05)	0.039
3 months	7.92	−1.30 (−2.23 to −0.36)	0.0072

*PCS*			
Baseline	18.63	NA	NA
2 weeks	15.01	−3.69 (−5.95 to −1.42)	0.0022
3 months	14.94	−3.62 (−5.92 to −1.33)	0.0029

*FFMQ*			
Baseline	128.66	NA	NA
2 weeks	135.63	7.03 (3.16 to 10.89)	0.0005
3 months	135.68	6.98 (3.04 to 10.91)	0.0006

*NPSI*			
Baseline	0.38	NA	NA
2 weeks	0.32	−0.06 (−0.10 to −0.02)	0.007
3 months	0.32	−0.06 (−0.10 to −0.01)	0.0104

^∗^An increase in score indicates a benefit for the SF-12, FFMQ variables, and the PGIC score, while a decrease in score indicates a benefit for all other measures (BPI, PHQ-9, POMS, PCS, and NPSI).

## Data Availability

The data used to support the findings of this study are available from the corresponding author upon request.

## Additional Points

*Key Message*. This article evaluates the impact of a mindfulness-based stress reduction intervention as compared to the waitlist control group among breast cancer survivors living with chronic neuropathic pain. We found that both groups reported similar scores three months after receiving the intervention, for all outcomes of interest. Due to reviews that highlight concerns that nonsignificant results of mindfulness-based stress reduction are often unpublished, the current study findings are important to disseminate.

## Supplementary Materials

Supplementary Materials CONSORT-SPI 2018 checklist.
